# Acute macular neuroretinopathy associated to dengue disease

**DOI:** 10.1016/j.ajoc.2022.101474

**Published:** 2022-03-31

**Authors:** Amir Translateur, Mario Perez-Rueda

**Affiliations:** a Ophthalmology Resident, Escuela Superior de Oftalmología, Instituto Barraquer de América, Bogotá, D.C., Colombia; b Neuro-Ophthalmology Department, Clínica Barraquer, Bogotá, D.C., Colombia

**Keywords:** Dengue, Neuroretinopathy, Vascular, Immune, Macula

## Abstract

**Purpose:**

To highlight an interesting case of Acute Macular Neuroretinopathy (AMN) in the context of dengue disease.

**Observations:**

A 70 year old woman from Ibagué, Colombia developed AMN during her hospitalization for dengue illness with warning signs. Her initial ophthalmic evaluation revealed a deep visual loss with no noticeable biomicroscopic findings and altered outer retinal layers in her macular optical coherence tomography (OCT). After a five year follow up, she maintains a poor visual acuity.

**Conclusions and Importance:**

There are few reported cases of AMN associated to dengue. This case report highlights the common physiopathological pathways between dengue and AMN, and the crossroads between vascular, infectious, and immune disorders.

## 1 Introduction

Dengue virus is part of the *Flaviviridae* family and there are four serotypes which are named DEN1 to DEN4. Patients may present a broad spectrum of clinical signs and symptoms that range from unspecific malaise and fever to severe dengue. The preferred vector of the disease is the *Aedes* sp. family of mosquitoes, especially *A. aegypti*, and *A. albopictus* (in order of decreasing frequency).^1^ There are 58.4 million yearly dengue diagnosis, and about 10 thousand deaths. Herein, the economic burden of the disease is estimated to be of $39.3 billion dollars.^2^

The transmission of this disease is the result of a complex interaction between people, mosquitoes, the virus itself, and environmental factors. The trend of global mobilization is an important factor in serotype amplification and viral heterogeneity. The aforementioned extent of clinical panoramas is due to the genetic diversity of the four types of viruses (which share between 60 and 75% of their genome), and the host's immune response.^3^

The specific pathophysiologic process that leads to ocular disease has not been described thus far, yet there is a high degree of suspicion that points to immune mediated mechanisms. The most common ophthalmologic symptoms of dengue are blurry vision, central scotomas and ocular pain. The most common signs in the posterior segment are maculopathy due to hemorrhages, macular edema, and optic neuropathy, in decreasing order.^4^ There are few case reports on Acute Macular Neuroretinopathy (AMN) in the context of dengue disease, which makes this event particularly noteworthy.

## 2 Materials and methods

This case report presents the five year long evolution on a case that was remitted to our clinic. Our patient was studied using macular OCTs, visual evoked potentials, magnetic resonance imaging (MRI), optometry examinations, full field electroretinograms (ERG), fluorescein angiography, ophthalmology consultations, Humphrey visual fields, and multifocal electroretinograms. Diagnosis of dengue disease can be done in endemic areas in accordance to the criteria outlined by the World Health Organization. Dengue may indeed mimic a variety of other diseases, or even symptoms of other flaviviruses, but these are uncommon in this area. The diagnosis of dengue disease is clinical and varies according to the region of the world, and no biomarker is essential for its diagnosis nor has a definitive predictive value.^5^, ^6^, ^7^

## 3 Clinical case

**Fig. 1:**
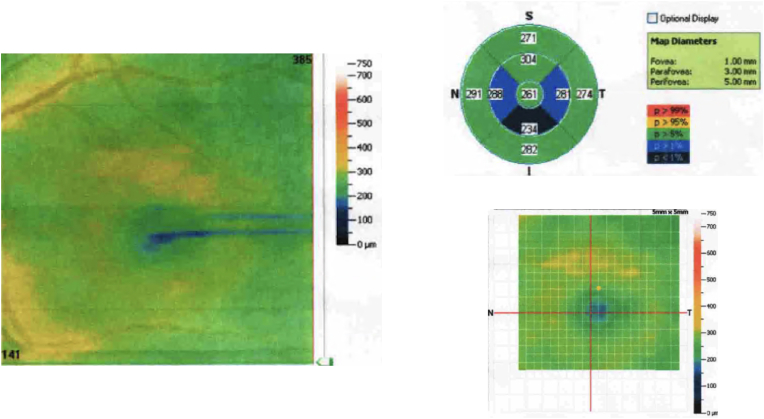
Macular OCT showing parafoveal thinning.

A 70 year old hispanic woman from Ibagué, Tolima, Colombia consulted our neuro-ophthalmology department and reported bilateral vision loss of acute onset that had evolved for the past three months, first noted during an episode of dengue that required hospitalization between December 14 to 19, 2014 for acute dengue disease with warning signs in an endemic area. Her warning signs included hemoconcentration with rapid platelet loss that reached a nadir of 18000/mm^3^, as well as abdominal pain, diarrhea, and lethargy.^8^ A month after discharge from the hospital where she was treated for her general dengue symptoms she was first seen by a local ophthalmologist, whose eye exam was unremarkable yet he requested left eye macular OCT due to her symptoms which showed parafoveal thinning (see Fig. 1).

During her first optometrical and ophthalmologic consultation at the Clínica Barraquer de America, on March 2, 2015, a best-corrected visual acuity (BCVA) of 20/200 and 20/400 was reported in her right and left eyes, respectively. The full ophthalmologic examination was almost entirely normal without any notable features in the anterior and posterior segment. Due to the patient's clinic and symptoms, complementary tests were performed. A left relative pupillary afferent defect (RPAD) was quantified in 0.3–0.6 logU. Ishihara tests yielded 4/14 in the right eye and no control for the left eye.

Further studies included electrophysiological exams and neuroimaging. Contrasted MRIs of the brain and orbit showed no lesions or signs that explained visual loss. Visual field tests demonstrated a paracentral supratemporal scotoma in the right eye and paracentral superior scotoma in the left eye. Visual evoked potentials (VEP) showed bilateral alterations in the retino-cortical transmission. The multifocal electroretinogram (mfERG) evidenced an important macular function alteration. No pathological findings were reported in the full field electroretinogram in both eyes. Fluorescein angiography of both eyes was unremarkable.

For the following 4 years the patient did not show up for her scheduled appointments, until April 2019, where visual acuity was reassessed without significant changes with a BCVA of 20/150+ in her right eye and 20/400 in her left eye. A full ophthalmologic examination was performed once again and was found normal without pathological findings in both anterior, and posterior segments of both eyes. A discrete improvement was found in the Ishihara test with results 11/14 for her right eye and 5/14 for her left eye. RPAD was mildly positive in her left eye.

**Fig. 2:**
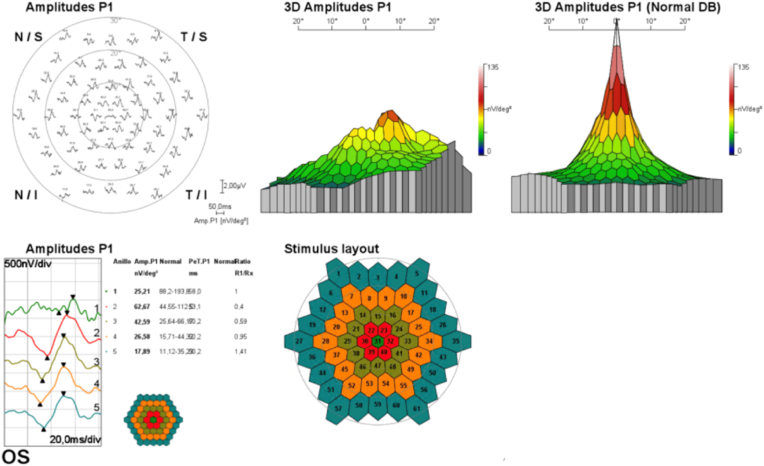
mfERG showing a severe functional depression in her left eye.

Follow up visual field tests, OCT and electrophysiological studies were requested. The 30–2 visual field demonstrated a paracentral inferotemporal, and superior depression in her right eye, and a paracentral superotemporal depressed area in the left eye. The 10–2 visual field showed an area of depression in the superotemporal quadrant and inferonasal area in her right eye, and the left eye showed an area of superotemporal, and supero-nasal depression. The new mfERG showed integrity of the central photoreceptors in the right eye, but a severe functional depression in her left eye (see Fig. 2).

**Fig. 3:**
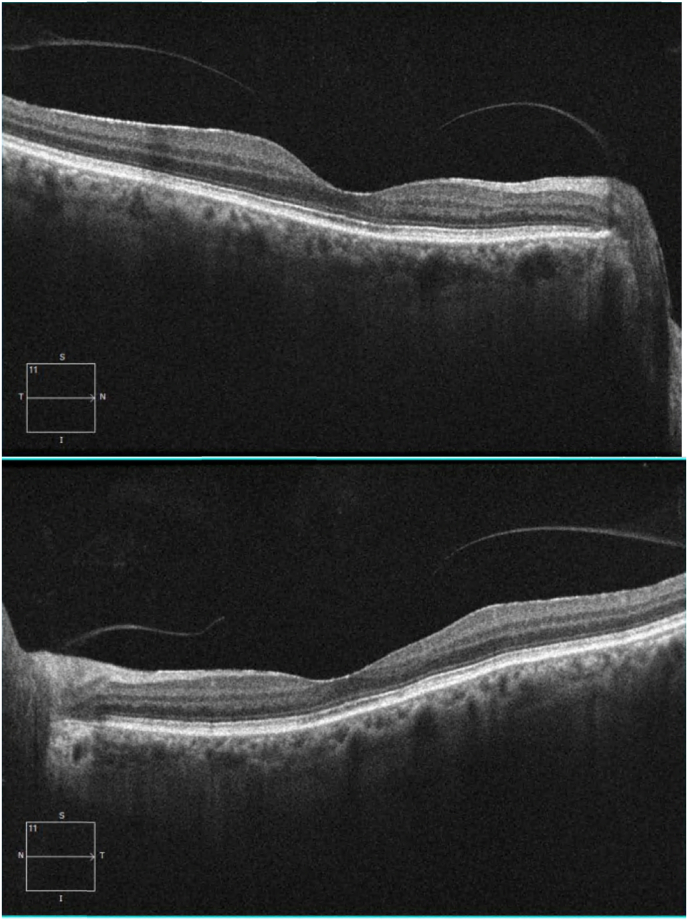
(a) Macular OCT showing a foveal disruption of the IZ in the right eye. (b) Macular OCT similar to the previous results in the left eye.

The macular OCT evidenced disruption of the interdigitation zone (IZ) in both eyes with a diminished central macular thickness (see Fig. 3a and b). Herein, the diagnosis of acute macular neuroretinopathy was made, and the patient was referred to a low vision specialist.

## 4 Discussion

Acute Macular Neuroretinopathy (AMN) is an unusual retinal disease of unknown etiology that is clinically characterized by wedge shaped reddish-brown macular lesions with their tip facing the fovea centralis. Most patients refer scotomas, a decreased visual acuity (VA), myodesopsia or metamorphopsia. More than 80% of patients have a VA of 20/40 or better during the disease's onset. Patients are characteristically female (M:F ratio = 0.16:1), around 30 years old, and of Caucasian origin.^8^

The most common visual field defect is one or more paracentral scotomas. Fluorescein angiographies are likely to be normal, and out of those described as abnormal the most frequent finding is hypofluorescence of the lesions in both early and late stages of the exam. Spectral Domain OCT (SD-OCT) commonly shows outer retinal layer anomalies such as an interrupted ellipsoid zone (EZ), a hyperreflective inner nuclear layer (INL), thinning of the INL, and in a very small percentage a hyporreflective retinal pigment epithelium (RPE)-photoreceptor junction. Patients with AMN are commonly studied with electrophysiologic tests. Those who have been assessed with full field electroretinograms (ERG) often have normal results. However, mfERGs tend to have diminished amplitudes in all waves.^8^

An analysis of risk factors points to a probable retinal vascular etiology. An article published by Monk and peers in 2015 proposed a series of probable AMN causes such as dengue, anemia, thrombocytopenia, leukemia, and Valsalva manoeuvre. Besides the well known mechanisms of hypovolemia and dehydration, these authors propose other pathophysiologic routes such as hyperviscosity due to leukocytosis, an increased capillary permeability, endothelial dysfunction, hemorrhagic diathesis, and pre-capillary occlusions due to immune complex deposits. AMN lesions tend to occur between the outer nuclear (ON), and outer plexiform (OP) layers, which suggests that the deep capillary plexus of the retina might be involved.^8^

The previously mentioned retinal signs of dengue seem to be compatible with its known pathophysiology which involves thrombocytopenia and the resulting hemorrhagic diathesis.^4^ However, it is now known that the retinal damage could be immunogenic and this is related to this specific case, which presents without bleeding in the posterior pole. Viral antigens recognized by cytotoxic T cells in infected monocytes elevate pro-inflammatory and vasoactive cytokine titers which lead to capillary leaking, macular edema, and its associated vascular dysfunction.^9^ In our patient's SD-OCT there is a sectoral atrophy of the rod and cone layer without edema or hemorrhagic maculopathy, which points to a probable vascular dysfunction of immune etiology.

OCTs are not only a good diagnostic method for patients with AMN, but it's also proven to be an effective follow-up, and prognostic tool in dengue-related maculopathy.^8^ Teoh, and peers, classify dengue maculopathy in three distinct patterns: diffuse macular thickening, cystoid macular edema, and third, foveolitis. In a series of 41 patients, all those who belong to the third group (foveolitis) had a central or paracentral scotoma in a two-year follow-up. Furthermore, 96% presented with a BCVA of 20/80 or worse at diagnosis, where only 8% maintained this visual acuity after two years, whereas most of the patients in the other two groups improved.^10^ Though our patient did not have cystic foveolitis (as the study describes) she does have an alteration that is exclusively confined to the fovea, so her clinical behaviour could be expected to be similar to those displaying such pattern. A limitation of this case is clearly the time between her acute illness and the time of presentation at the Clinic, which was about three months later. During this period, either during biomicroscopic or macular OCT examination, foveolitis could've been present. More recently, Agarwal, and peers have described a series of 32 eyes with dengue-induced inflammatory, ischemic foveolitis and outer maculopathy (DII-FOM) which is characterized by few posterior vitreous cells, altered foveal reflex, ischemia of retinal layers and outer retinal changes presented within a couple of weeks of fever onset.^11^ Some of these changes could have been noticed in our patient if she had promptly consulted an ophthalmologist.

## 5 Conclusion

There are very few cases reported of dengue-associated AMN. This disease is particularly challenging since it is not easily diagnosed through a complete ophthalmologic examination and requires a high degree of suspicion when analyzing OCTs. Hence, the authors of this article find it a particularly educational case that provides an interesting clinical challenge and highlights interesting aspects of immune, infectious, and vascular diseases of the retina.

## Patient consent

Written consent to publish this case has not been obtained. This report does not contain any personal identifying information.

## Funding

No funding or grant support.

## Authorship

All authors attest that they meet the current ICMJE criteria for Authorship.

## Declaration of competing interest

**T**he following authors have no financial disclosures: AT, MP.
